# 

**Published:** 1912-06

**Authors:** 


### CONTENTS.

ORIGINAL COMMUNICATIONS:—

Etiology, Pathology and Treatment of Pellagra. By Geo. C. Mizell, M. D., Atlanta, Ga. ........ 112
A Brief History of the Medical Milk Commission in the United States. By C. A. Rhodes, M. D., Atlanta, Ga. ........ 125
Some Observations on the Use of Obstetrical Forceps. By H. R. Donaldson, A. B., M. D. ........ 129
Some Suggestions That Aid in Surgical Success. By R. R. Kime, M. D., Atlanta, Ga. ........ 132
The Treatment of Eclamptic Convulsions. By James R. McCord, M. D., Atlanta, Ga. ........ 135
The Tubercular Negro—An Unsolved Problem—By W. C. Bryant, Clarksville, Ga. ........ 140
Hemorrhagic Diseases of the Kidney—Report of a Case of Probable Renal Varix. By Edgar G. Ballanger, M. D., and Omar F. Elder M. D., Atlanta, Ga. ........ 144
EDITORIAL ........ 149
NEWS AND NOTES ........ 151
BOOK REVIEWS ........ 168

